# 12,13-diHOME and noradrenaline are associated with the occurrence of acute myocardial infarction in patients with type 2 diabetes mellitus

**DOI:** 10.1186/s13098-023-01115-9

**Published:** 2023-06-29

**Authors:** Ning Cao, Yichun Wang, Boyi Bao, Man Wang, Jiayu Li, Wenxi Dang, Bing Hua, Lijin Song, Hongwei Li, Weiping Li

**Affiliations:** 1 Department of Cardiology, Cardiovascular Center, Beijing Friendship Hospital, Capital Medical University, 95 Yongan Road, Beijing, 100050 China; 2 Beijing Key Laboratory of Metabolic Disorder Related Cardiovascular Disease, Beijing, China; 3 Department of Gastroenterology, Peking University Third Hospital, Beijing, 100191 China

**Keywords:** Acute myocardial infarction, Diabetes mellitus, 12,13-dihydroxy-9Z-octadecenoic acid, Noradrenaline, Metabolomics

## Abstract

**Background:**

Acute myocardial infarction (AMI) is the most prevalent cause of mortality and morbidity in patients with type 2 diabetes mellitus (T2DM). However, strict blood glucose control does not always prevent the development and progression of AMI. Therefore, the present study aimed to explore potential new biomarkers associated with the occurrence of AMI in T2DM patients.

**Methods:**

A total of 82 participants were recruited, including the control group (n = 28), T2DM without AMI group (T2DM, n = 30) and T2DM with initial AMI group (T2DM + AMI, n = 24). The untargeted metabolomics using liquid chromatography-mass spectrometry (LC–MS) analysis was performed to evaluate the changes in serum metabolites. Then, candidate metabolites were determined using ELISA method in the validation study (n = 126/T2DM group, n = 122/T2DM + AMI group).

**Results:**

The results showed that 146 differential serum metabolites were identified among the control, T2DM and T2DM + AMI, Moreover, 16 differentially-expressed metabolites were significantly altered in T2DM + AMI compared to T2DM. Amino acid and lipid pathways were the major involved pathways. Furthermore, three candidate differential metabolites, 12,13-dihydroxy-9Z-octadecenoic acid (12,13-diHOME), noradrenaline (NE) and estrone sulfate (ES), were selected for validation study. Serum levels of 12,13-diHOME and NE in T2DM + AMI were significantly higher than those in T2DM. Multivariate logistic analyses showed that 12,13-diHOME (OR, 1.491; 95% CI 1.230–1.807, *P* < 0.001) and NE (OR, 8.636; 95% CI 2.303–32.392, *P* = 0.001) were independent risk factors for AMI occurrence in T2T2DM patients. The area under receiver operating characteristic (ROC) curve (AUC) were 0.757 (95% CI 0.697–0.817, *P* < 0.001) and 0.711(95% CI 0.648–0.775, *P* < 0.001), respectively. The combination of both significantly improved the AUC to 0.816 (95% CI 0.763–0.869, *P* < 0.001).

**Conclusions:**

12,13-diHOME and NE may lead to understanding the possible metabolic alterations associated with AMI onset in T2DM population and serve as promising risk factors and therapeutic targets.

**Supplementary Information:**

The online version contains supplementary material available at 10.1186/s13098-023-01115-9.

## Background

The prevalence of type 2 diabetes mellitus (T2DM) has steadily increased worldwide. People with diabetes comprise 8.8% of the world’s population, and the International Diabetes Federation (IDF) predicts that the number of diabetes cases will increase to 642 million by 2040. The prevalence of acute myocardial infarction (AMI) is higher in adults with diabetes compared to that in adults without T2DM; AMI is the most common cause of mortality and morbidity in this population [1].

The importance of intensive glycemic control for protection against microvascular complications and cardiovascular disease (CVD) in people with T1DM is well-established [2]. However, its role in reducing cardiovascular risk has not been elucidated in people with T2DM. Strict blood glucose control does not always prevent the development and progression of AMI [3], and a considerable proportion of T2DM patients did not develop AMI during a 7-year follow-up period [4, 5]. The influence of hyperglycemia and insulin resistance on the onset of AMI, and whether they could be symptoms of T2DM, but might not necessarily contribute to its pathogenesis, is not clear. In addition to hyperglycemia, the deregulation of other cellular activities, such as the generation of reactive metabolites, could contribute to the development of AMI.

Metabolomics aims to measure metabolite concentrations in cells, tissues, organs, and biological systems to systematically investigate the chemical processes involved in metabolism. Recently, it has become a promising diagnostic and prognostic tool. Metabolites are particularly attractive as biomarkers for metabolic diseases because their accumulation or deficiency frequently causes various disease. T2DM is well-suited for metabolomic studies because it is a polygenic metabolic disease with significant contributions from behavioral and environmental factors [6]. There is a growing body of literature describing metabolomic profiles associated with T2DM in both patients and animal models. However, there is a lack of metabolomic studies in T2DM patients with initial AMI.

Therefore, it is necessary to assess the underlying mechanisms and new biomarkers for identifying T2DM patients at a high risk of AMI. This study aimed to investigate the association between circulating metabolites and possibly involved metabolic pathways and the occurrence of AMI in T2DM patients by using liquid chromatography–mass spectrometry (LC–MS) metabolomics approach. The findings would provide insights into the novel targets for prevention of AMI.

## Methods

### Study population

This retrospective discovery phase study was based on the Cardiovascular Center Beijing Friendship Hospital Database Bank (CBD Bank) from March 2017 to May 2020. 30 patients each group from all the enrolled patients were selected through random numbers using the Microsoft Office Excel (office 2021). At the same time, abnormal samples were removed based on the principal component analysis (PCA) score plot. Eventually, 24 T2DM patients presenting with their first ST-elevation myocardial infarction (STEMI) within the first 12 h of the onset of chest pain were enrolled from the Department of Cardiology of Beijing Friendship Hospital. 30 cases of T2DM patients without AMI, who exhibited negative results or < 50% obstruction on coronary artery CT or coronary angiography, and 28 healthy control subjects were enrolled during this period. The exclusion criteria were as follows: (1) previous history of CVD, valvular disease, congenital heart disease, hypertension, chronic kidney disease, stroke, and hyperlipidemia; (2) presence of acute infection, severe hepatic dysfunction, tumor, rheumatic immune disease. All subjects in the three groups were matched for age, sex, and body mass index (BMI).

The validation phase study was performed on another independent population. All T2DM participants who met the inclusion and exclusion criteria were enrolled according to combined initial AMI or not. The validation study included 126 T2DM patients without AMI (T2DM group) and 122 T2DM patients with their first STEMI (T2DM + AMI group). They were recruited from March 2017 to July 2021. The exclusion criteria were as follows: (1) previous history of CVD, severe valvular disease, or congenital heart disease; (2) severe renal dysfunction (serum creatinine > 3 mg/dl); (3) the presence of acute infection, severe hepatic dysfunction, tumor, or rheumatic immune disease.

The study protocol was approved by the Institutional Review Board of Beijing Friendship Hospital and conducted in accordance with the Declaration of Helsinki.

### Data collections and definitions

Patient demographics, medical history, therapy, laboratory data, and echocardiographic and angiographic results were collected and verified using an electronic medical record system.

The criteria for T2DM include: (1) previously diagnosed T2DM under treatment with antidiabetic medication; (2) the typical symptoms of T2DM with a fasting plasma glucose (FPG) ≥ 7.0 mmol/L, and/or random blood glucose (RBG) ≥ 11.1 mmol/L, and/or 2-h plasma glucose level following oral glucose tolerance test (OGTT) ≥ 11.1 mmol/L. STEMI was defined as ischemic symptoms with new ST-segment elevation and increased cardiac troponin I (cTnI) or troponin T (cTnT) levels above the 99^th^ percentile upper reference limit.

### Sample preparation for metabolomics

Peripheral venous blood samples were collected from all subjects on admission. The sample was centrifuged at 1000 g at 4 ℃ for 15 min; the supernatant serum was obtained, aliquoted, and stored at −80 °C until liquid chromatography-mass spectrometry (LC/MS) analysis. Hemolytic or chylous samples were excluded from analysis. Before analysis, serum samples were thawed at 4 ℃. After vacuum drying and redissolving, each sample was mixed with internal standard solution and used for LC/MS detection and quality control (QC). For semi-quantitative detection of metabolites, the supernatant of the standard curve correction solution was obtained by mixing the serum sample, vortexing, and centrifuging at 4000 g at 4 °C for 10 min [7]. The detailed description of the LC–MS sample preparation process was shown in Additional file 1.

### Metabolomics analysis using LC–MS

To obtain a complete metabolic profile, untargeted metabolomics analysis was conducted using UPLC-MS. Chromatographic separation was performed in a Thermo Vanquish system equipped with an ACQUITY UPLC^®^HSS T3 (150 × 2.1 mm, 1.8 µm, Waters, USA) column maintained at 40 ℃. The temperature of the autosampler was 8 ℃. ESI–MS experiments were performed on a Thermo Q Exactive mass spectrometer with a spray voltage of 3.5 kV and −2.5 kV, in positive and negative modes, respectively. The capillary temperature was 325 ℃. The analyzer scanned over a mass range of m/z 81–1000 for the full scan at a mass resolution of 70,000. The normalized collision energy was 30 eV [8]. The detected ions were subjected to isotopic calibration using the accurate masses of the reference standards. (Positive mode: Val-^13^C, Choline-d9, Phe-^13^C, L-carnitine-d3, and betaine-d9; negative mode: F-^13^C, Val-^13^C, Phe-^13^C, U-2-^13^C, and VB3-d4). The additional description of the LC–MS method and the QC results were shown in Additional file 1.

### Data processing and metabolites identification

The raw data were converted to mzXML format using ProteoWizard (v3.0.8789). Identification, filtration, and alignment of the peaks were performed using the R-package XCMS (R-v3.3.2). After batch normalization, the mass spectrometry data were used for the relative quantification of the metabolites in the sample solution using a linear fitting equation and isotopic internal standard preference. The metabolites were identified using databases including Metlin (http://metlin.scripps.edu), MONA (http://mona.fiehnlab.ucdavis.edu//), and the metabolome database constructed using BioNovoGene (Suzhou, China), with a mass accuracy of 15 ppm. Agglomerate hierarchical cluster analysis was performed using the R-package heatmap (R-v3.3.2). The metabolic pathways of the altered metabolites were integrated using the Kyoto Encyclopedia of Genes and Genomes (KEGG) pathway database (http://www.genome.jp/kegg/) and MetaboAnalyst (http://www.metaboanalyst.ca/).

### Enzyme-Linked-Immunosorbent-Assay (ELISA)

The serum levels of 12,13-diHOME, noradrenaline, and estrone sulfate in the verification cohort were determined using commercial ELISA kits purchased from Cayman chemical (No. 501720, Ann Arbor, Michigan USA), Abnova (KA1877, Taiwan, China), and Thermo Scientific (EIA17E3S, Waltham, MA, USA). Briefly, the patient’s serum obtained during admission was thawed from -80℃ to room temperature. The procedures were performed according to the manufacturer’s instructions, and all sample concentrations were within the standard curve range.

### Statistical analysis

Data are expressed as mean ± standard deviation (SD) or median (interquartile range) for continuous variables and as numbers (percentages) for categorical variables. Shapiro–Wilk test was used for data normality testing. Continuous data from two groups were compared using Student’s *t*-test or Wilcoxon rank sum test. Intergroup comparisons of continuous variables were performed using One-way ANOVA or Kruskal–Wallis rank sum test with the least significant difference (LSD) post hoc test for continuous variables and Pearson’s chi-square test or Fisher’s exact test for categorical variables, where appropriate.

For the efficient identification of differences in the metabolic profiles between the groups, the PCA and the orthogonal projection to latent structure-discriminant analysis (OPLS-DA) model was applied using the R software (version 3.3.2). The variable importance in the point (VIP) value of each variable in the model was calculated to indicate its contribution to the classification. A higher VIP value indicates a more vital contribution to the discrimination among the groups. VIP > 1.0 and *P* < 0.05 were considered significant. Univariate and multivariate logistic regression analyses were used to determine the independent risk factors for AMI in all participants with T2DM. Odds ratios (OR) and 95% confidence intervals (CI) were calculated. Receiver operating characteristic (ROC) curve analysis was performed to assess the clinical performance of metabolites, and the area under the curve (AUC) was evaluated.

All statistical analyses were performed using the Statistical Package for the Social Sciences (SPSS) version 26.0 (IBM Inc., Armonk, NY, USA). Statistical significance was defined as a two-tailed p-value of < 0.05.

## Results

### Baseline characteristics in the discovery phase study

**Fig. 1:**
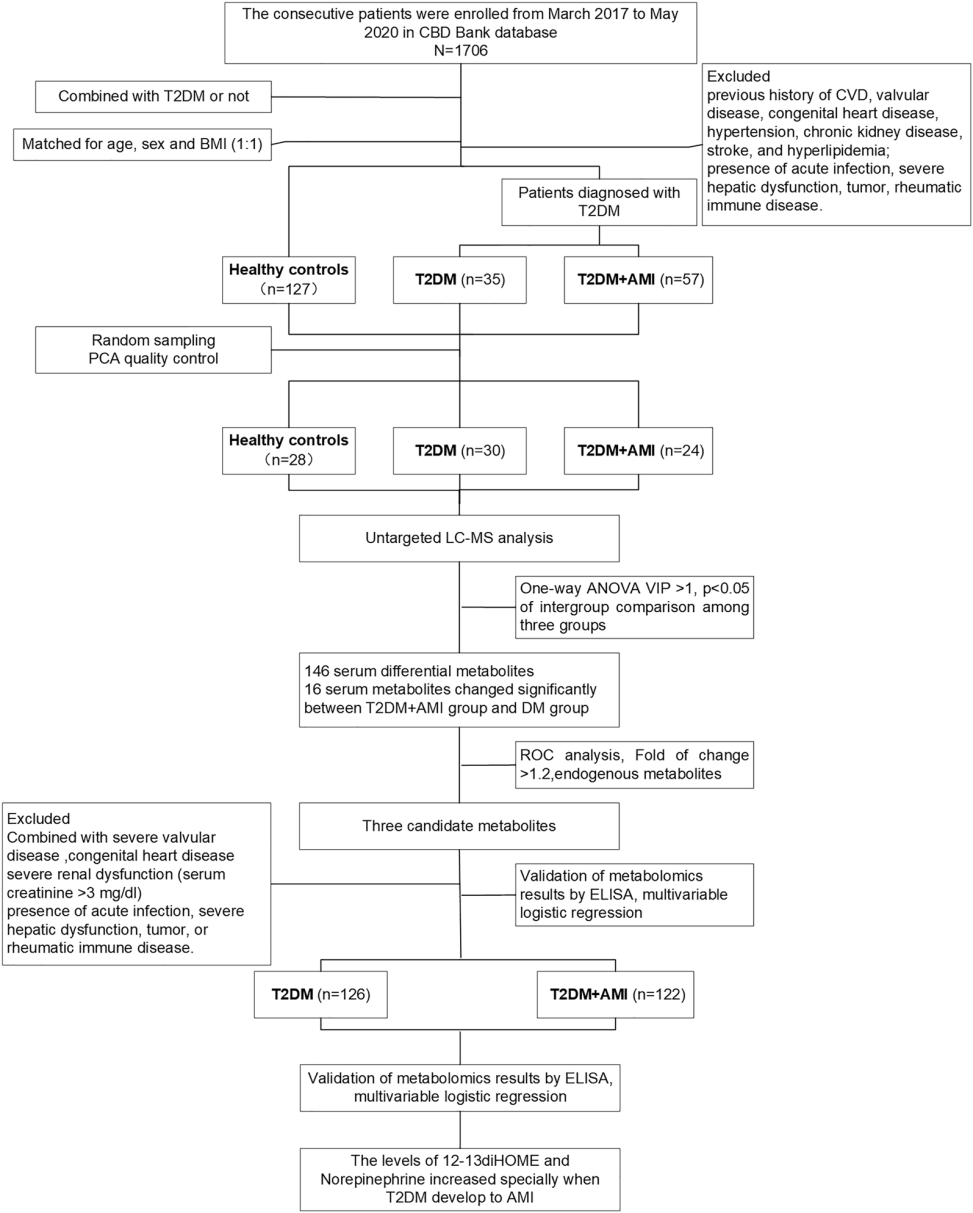
Flow diagram of overview of the study. *T2DM* diabetes mellitus, *AMI* acute myocardial infarction, *BMI* body mass index, *LC–MS* liquid chromatography-mass spectrometry, *One-way ANOVA* one-way analysis of variance, *VIP* variable important in projection, *P* p-value, *ROC* receiver operating characteristic, *ELISA* enzyme-linked immunosorbent assay

An overview of the study design is shown in Fig. 1. After validation of the kit plate using QC samples, 82 serum samples from 28 normal control subjects, 30 T2DM patients, and 24 T2DM + AMI patients were included in the untargeted metabolomics analysis.

Baseline characteristics and laboratory data of the study population are presented in Table 1. Compared to the control subjects, the patients in T2DM group and T2DM + AMI group had lower high-density lipoprotein (HDL) levels and higher hypersensitive C-reactive protein (hs-CRP), hemoglobin A1c (HbA1c) and fasting blood glucose levels. There were no significant differences in age, sex, body mass index (BMI), smoking history, serum triglyceride (TG), total cholesterol (TC), low-density lipoprotein cholesterol (LDL-C), or creatinine (Cr) among the three groups.

**Table 1:** Baseline clinical characteristics of the population used for the untargeted metabolomic analysis

	Control (n = 28)	T2DM (n = 30)	T2DM + AMI (n = 24)	*P* value
Age, years	58.89 ± 9.7	59.47 ± 9.4	62.71 ± 11.3	0.353
Male gender, n (%)	14 (50.0)	15 (50.0)	12 (50.0)	1.00
BMI, Kg/m^2^	24.80 ± 3.54	26.03 ± 3.17	25.29 ± 3.26	0.370
Smoking, n (%)	13 (46.4)	11 (36.7)	12 (50)	0.585
Duration of diabetes, years	–	1.0 (1.0–4.0)	1.0 (1.0–2.3)	0.243
Laboratory values				
Cr, umol/L	64.10 (52.20–76.10)	62.30 (52.30–69.80)	67.90 (52.78–78.63)	0.358
TC, mmol/L	4.16 (3.70–5.02)	4.13 (3.54–4.75)	4.19 (3.50–4.56)	0.306
TG, mmol/L	1.00 (0.77–1.74)	1.33 (1.03–1.98)	1.52 (0.90–2.36)	0.118
HDL-C, mmol/L	1.25 (1.13–1.61)	0.98 (0.87–1.26)_	1.01 (0.85–1.29)	0.002
LDL-C, mmol/L	2.30 (1.87–2.72)	2.28 (1.94–2.75)	2.39 (1.80–2.82)	0.738
Hs-CRP, mg/L	0.63 (0.38–2.48)	1.08 (0.54–3.58)	8.67 (3.16–27.15)	< 0.001
HbA1c, %	5.50 (5.30–5.70)	6.50 (6.20–7.30)	7.50 (6.73–9.08)	< 0.001
Fasting glucose, mmol/L	4.85 ± 0.41	6.46 ± 1.34	10.07 ± 3.80	< 0.001
CK-MB, ng/ml	1.00 (0.60–1.30)	0.80 (0.50–1.10)	6.35 (2.15–23.45)	< 0.001
cTNI, ng/ml	0 (0–0)	0 (0–0)	0.41 (0.06–10.74)	< 0.001

Data are expressed as mean ± standard deviation,numbers (%) or median (interquartile range)

*T2DM* Type 2 diabetes mellitus, *AMI* acute myocardial infarction, *BMI* body mass index, *Cr* creatinine, *TC* total cholesterol, *TG* triglyceride, *HDL-C* high-density lipoprotein cholesterol, *LDL-C* low-density lipoprotein cholesterol, *hs-CRP* high-sensitivity C-reactive protein, *HbA1c* hemoglobin A1c, *CK-MB* creatine kinase isoenzyme, *cTNI* cardiac troponin I, *hs-CRP* high-sensitivity C-reactive protein, *CK-MB* Creatine Kinase MB Isoenzyme, *cTNI* cardiac troponin I

### Discrimination between T2DM patients with or without AMI and controls using serum untargeted metabolomics data

**Fig. 2:**
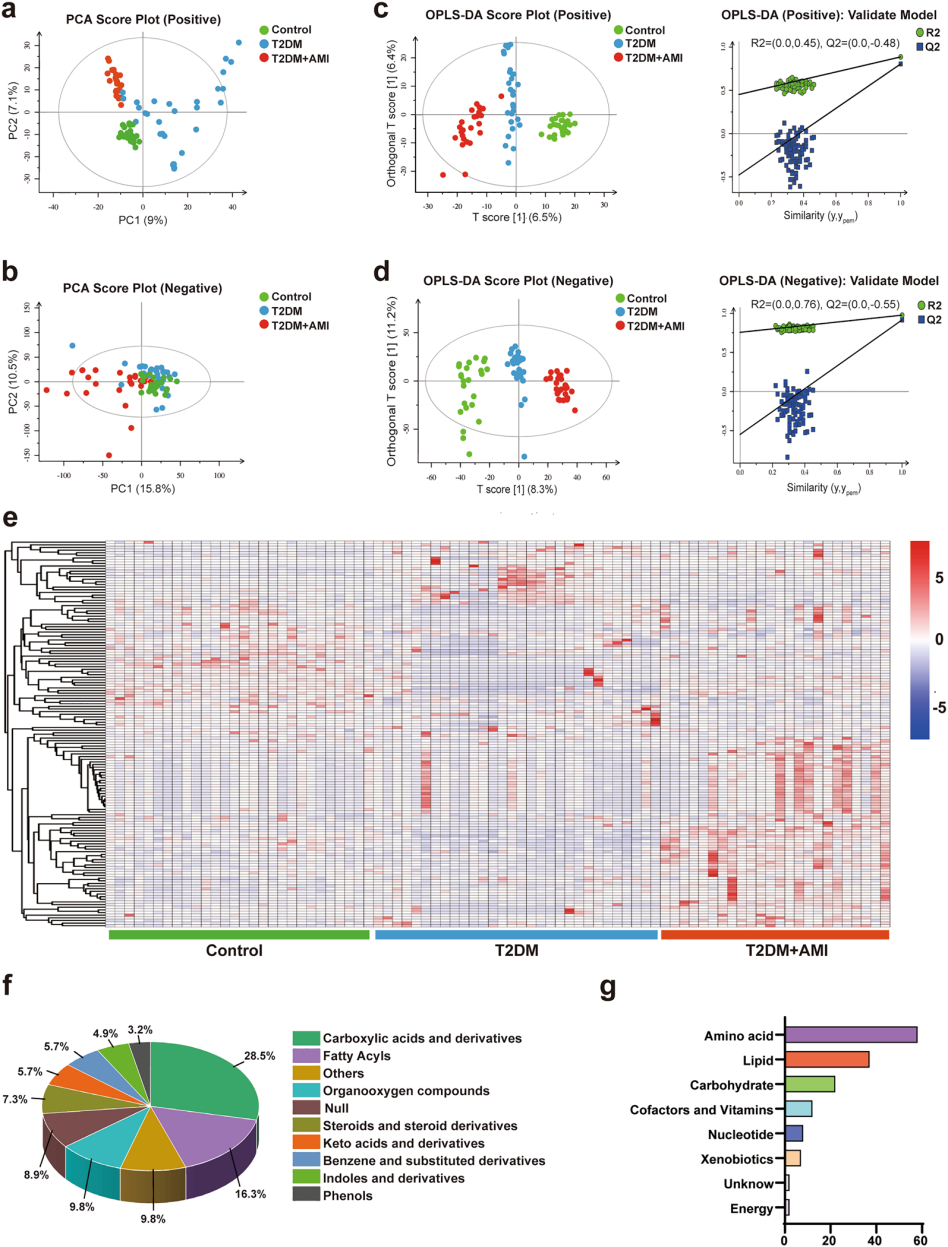
The 146 differential serum metabolites among control subjects and T2DM patients without or with AMI identified using untargeted metabonomic. **a** and **b**, The PCA score plots of differential serum metabolites either in positive or negative model from the untargeted metabonomic analysis. **c** and **d**, The OPLS-DA score plots and corresponding validation model of differential serum metabolites either in positive or negative model from the untargeted metabonomic analysis. **e**, The heatmap showing the levels of 146 differential metabolites. f and g, the differential serum metabolites classified according to the function (**f**) or category (**g**). Control: normal control subjects. n = 28; T2DM, patients with type 2 diabetes, n = 30; T2DM + AMI, type 2 diabetes patients with acute myocardial infarction (n = 24)

Untargeted metabolomics was used to record aligned metabolic features for each serum sample among the three groups in the MS positive and negative ion mode. After re-evaluation of the features, 222 metabolites were identified using mass chromatography (Additional file 2). The PCA and OPLS-DA results showed that the serum metabolites were different in both positive and negative models between the T2DM group or T2DM + AMI group and the control group (Fig. 2a–d). Among these metabolites, 146 differential metabolites between any two of the three groups were identified according to the contributions of OPLS-DA (VIP > 1, *P* < 0.05). The heatmap showed that the serum levels of 146 metabolites differed among the three groups (Fig. 2e).

Among these differential serum metabolites, 28.5% were carboxylic acids and derivatives and 16.3% were fatty acids (Fig. 2f). Amino acids and lipids were the two most essential subcategories (Fig. 2g). Detailed information regarding these metabolites is provided in Additional file 3.

### Selecting candidate biomarkers that differentiate patients with T2DM and those with T2DM and AMI

**Fig. 3:**
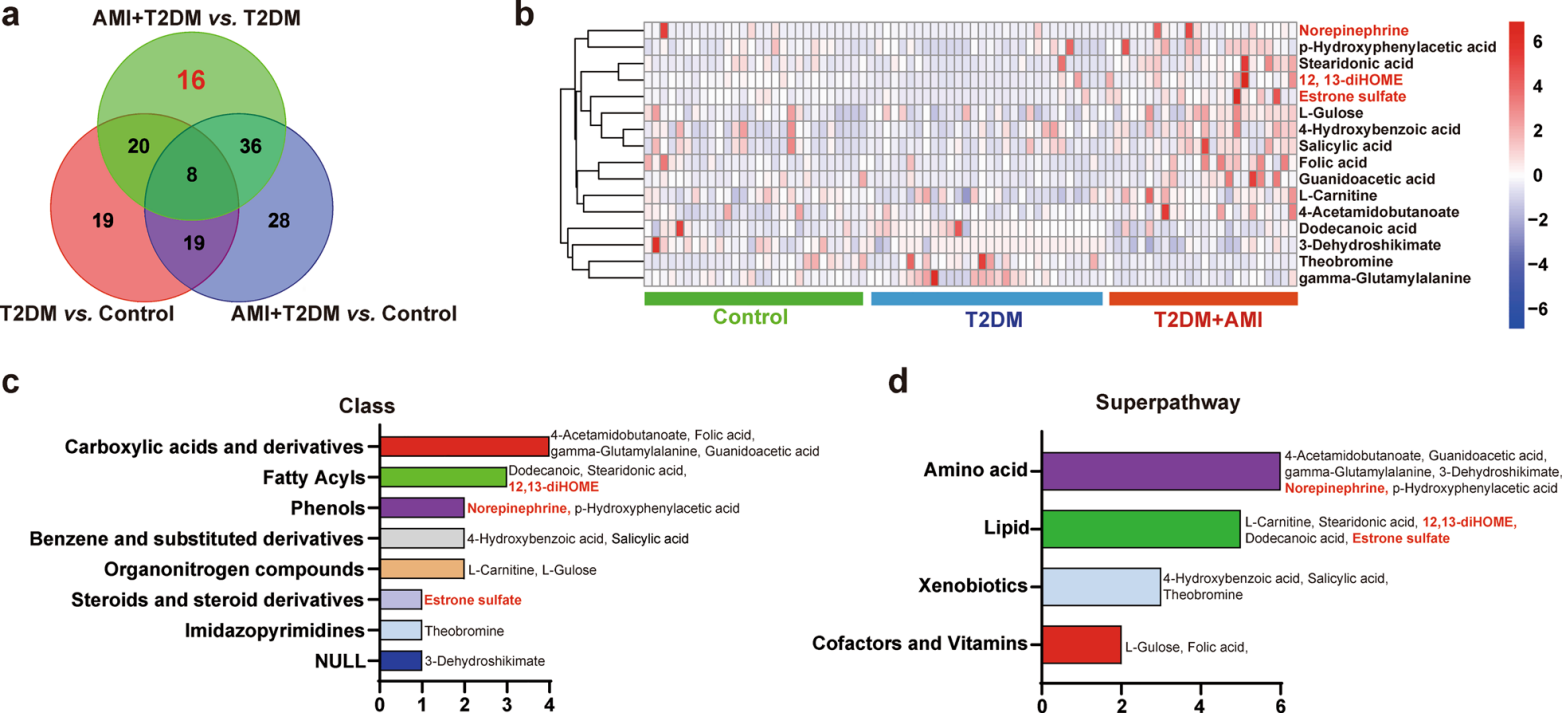
The serum levels of 16 specific metabolites were significantly different between T2DM patients with and without AMI. **a**, The Venn diagram indicating 16 specific differential metabolites between the T2DM and T2DM + AMI groups. **b**, The heatmap showing the concentrations of the 16 specific metabolites among the different groups. **c** and **d**, the 16 specific metabolites classified according to function (**c**) or category (**d**). Control: normal control subjects. n = 28; T2DM, patients with type 2 diabetes, n = 30; T2DM + AMI, type 2 diabetes patients with acute myocardial infarction (n = 24)

The criteria for further screening of specific serum metabolites among the differential metabolites between the T2DM group and T2DM + AMI group are illustrated in Fig. 2. The OPLS-DA contributions of candidate metabolites should be sufficiently high between the two groups (VIP > 1, *P* < 0.05). Accordingly, 16 specific differential metabolites between the T2DM group and T2DM + AMI group were identified from the differential metabolites among the three groups (Fig. 3a). The heatmap of the 16 metabolites is shown in Fig. 3b. Similar to that observed in the pairwise comparison in Fig. 2, these 16 metabolites majorly belonged to the class of carboxylic acid and derivatives and fatty acids. Notably, the super pathway also involved amino acids and lipids (Fig. 3c–d). Detailed information regarding these metabolites is provided in Additional file 4.

**Fig. 4:**
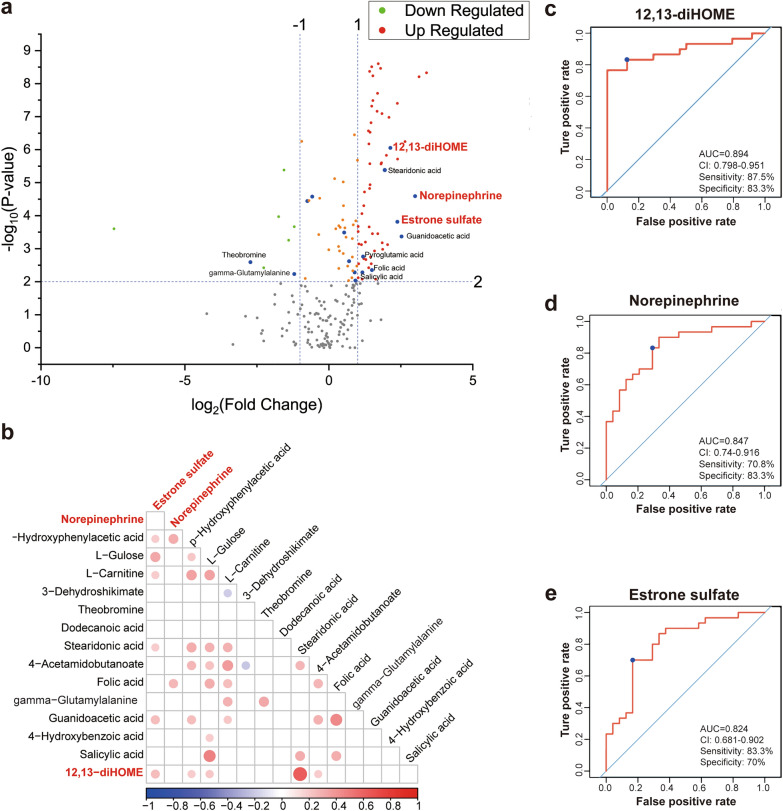
12, 13-diHOME, NE, and ES as candidate differential serum metabolites between the T2DM and T2DM + AMI groups. **a**, Volcano Plot of the 126 differential serum metabolites with the blue blots indicating the 16 specific serum metabolites differentially expressed between the T2DM and T2DM + AMI groups. **b**, Correlation analysis of the 16 specific serum metabolites in the T2DM + AMI group. **c-e**. ROC curves of 12,13-diHOME, NE, and ES for the identification of AMI in all T2DM patients analyzed using untargeted metabolomics. 12,13-diHOME, 12,13-dihydroxy-9Z-octadecenoic acid. NE, Norepinephrine. ES, Estrone sulfate. T2DM, patients with type 2 diabetes, n = 30; T2DM + AMI, type 2 diabetes patients with acute myocardial infarction (n = 24)

We then set the criteria for selecting candidate biomarkers. First, the serum metabolite levels should be significantly different between the T2DM group and T2DM + AMI group (fold change > 1.2, *P* < 0.05). Second, the candidate metabolites should be endogenous metabolites. Finally, the detection methods for candidate serum metabolites should be easy and well-established. Accordingly, 12,13-dihydroxy-9Z-octadecenoic acid (12,13-diHOME), noradrenaline (NE), and estrone sulfate (ES) were screened and highlighted in red in Figs. 3 and 4. Compared to that in the T2DM group, the serum levels of these three metabolites were significantly increased in the T2DM + AMI group (Fig. 4a). In addition, the level of 12, 13-diHOME was positively correlated with ES in the T2DM + AMI group, but there was no significant correlation between 12, 13-diHOME or ES with NE (Fig. 4b). ROC analysis demonstrated that all three metabolites had strong clinical values for AMI occurrence in patients with T2DM. The AUC of 12, 13-diHOME, NE and ES were 0.894 for, 0.847, and 0.824, respectively (Fig. 4c, d, and e).

### 12, 13-diHOME and NE are independently associated with the occurrence of AMI in patients with T2DM in the validation phase study

**Fig. 5:**
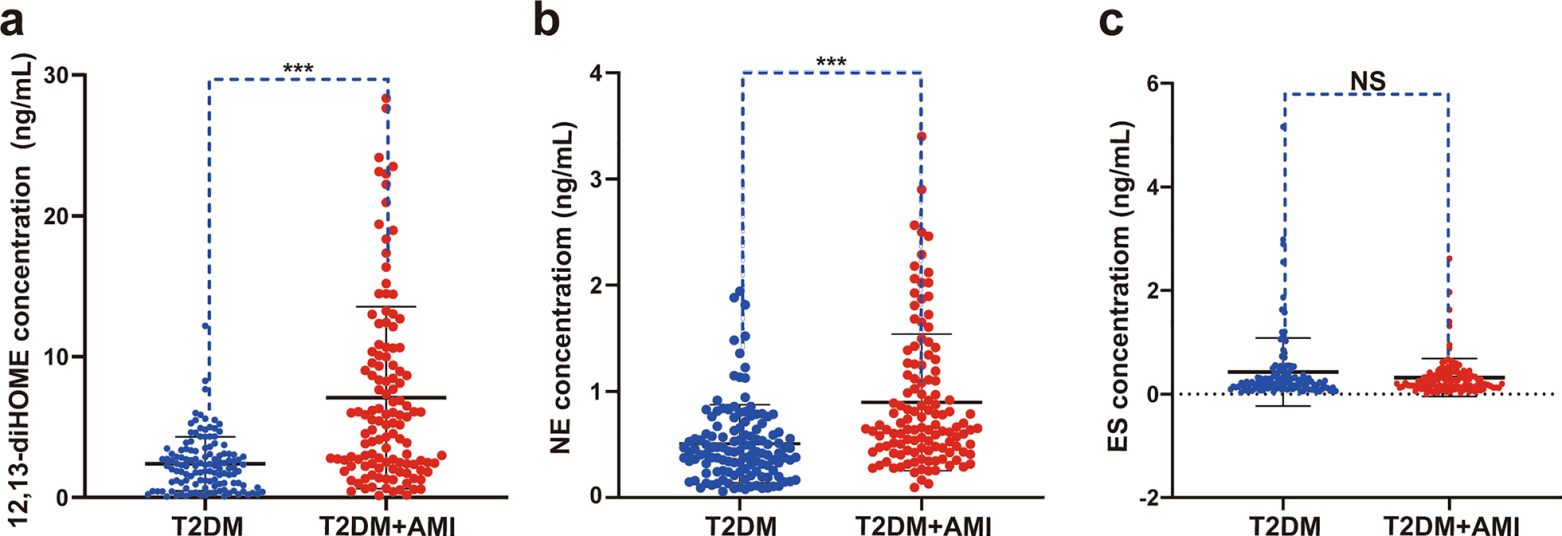
Serum levels of 12,13-diHOME, NE, and ES detected using ELISA in the T2DM and T2DM + AMI groups. **a-c**, serum concentrations of 12,13-diHOME, NE, and ES in T2DM patients with or without AMI. N = 122 in T2DM + AMI, N = 126 in T2DM, ^***^P < 0.001. 12,13-diHOME, 12,13-dihydroxy-9Z-octadecenoic acid. NE, Norepinephrine. ES, Estrone sulfate. T2DM, patients with type 2 diabetes

Another independent validation population was studied to identify whether the three selected metabolites could be used as new serum biomarkers for AMI in patients with T2DM. The validation phase included 248 participants, including 126 T2DM patients without CVD (T2DM group) and 122 T2DM patients with initial STEMI (T2DM + AMI group). The baseline clinical characteristics of the patients are shown in Table 2. Compared to the T2DM group, the T2DM + AMI group had a significantly higher proportion of males and previous history of smoking and hypertension, higher levels of fasting blood glucose, serum creatinine, TC, LDL-C, hs-CRP, HbA1c, CK-MB, and cTNI, lower HDL-C level and lower prior treatment with statins, angiotensin-converting enzyme inhibitor (ACEI)/angiotensin receptor blocker (ARB), and metformin. Consistent with the metabolomics data, the serum levels of 12, 13-diHOME, and NE detected by ELISA method were remarkably elevated in the T2DM + AMI group compared to that in the T2DM group (Fig. 5a, b). However, ES levels were similar between the two groups (Fig. 5c).

**Table 2:** Baseline clinical characteristics of the population used in the experimental verification groups

	T2DM (n = 126)	T2DM + AMI (n = 122)	*P* value
Age, years	63 (57–76)	62 (55–70)	0.684
Male gender, n (%)	54 (42.9)	89 (73)	< 0.001
BMI, Kg/m^2^	26.46 (23.64–28.58)	25.95 (24.17–27.71)	0.283
Smoking, n (%)	24 (19)	75 (61.5)	< 0.001
Hypertension, n (%)	40 (31.7)	76 (62.3)	< 0.001
Dyslipidemia, n (%)	78 (61.9)	65 (53.3)	0.169
Duration of diabetes, years	9.0 (3.0–15.0)	10.0 (3.3–15.0)	0.811
Laboratory values			
Cr, umol/L	62.05 (53.03–71.28)	73.40 (60.08–82.63)	0.001
TC, mmol/L	3.99 (3.31–4.60)	4.46 (3.85–5.04)	< 0.001
TG, mmol/L	1.38 (1.06–1.97)	1.36 (1.02–2.08)	0.697
HDL-C, mmol/L	1.06 (0.92–1.27)	0.96 (0.86–1.14)	0.014
LDL-C, mmol/L	2.20 (1.69–2.65)	2.65 (2.28–3.10)	< 0.001
Hs-CRP, mg/L	1.00 (0.46–2.97)	13.25 (2.57–29.29)	< 0.001
HbA1c, %	6.80 (6.20–7.50)	7.90 (6.95–9.13)	< 0.001
Fasting glucose, mmol/L	6.60 (5.46–7.54)	9.24 (6.71–12.34)	< 0.001
CK-MB, ng/ml	1.00 (0.70–1.55)	5.15 (1.93–20.35)	< 0.001
cTNI, ng/ml	0 (0–0)	0.67 (0.66–6.93)	< 0.001
Medical therapies before admission			
Antiplatelet agent, n (%)	32 (25.4)	24 (19.7)	0.281
Statin, n (%)	45 (35.7)	12 (9.8)	< 0.001
ACEI/ARB, n (%)	63 (50)	35 (28.7)	< 0.001
Diuretics, n (%)	14 (11.1)	7 (5.7)	0.129
Acarbose, n (%)	42 (33.3)	54 (44.3)	0.077
Metformin, n (%)	76 (60.3)	44 (36.1)	< 0.001
Sulfonylurea, n (%)	33 (26.2)	29 (23.8)	0.660
Insulin, n (%)	29 (23)	30 (24.6)	0.771
Insulin sensitizers, n (%)	10 (7.9)	4 (3.3)	0.112
DPP-4 inhibitors, n (%)	5 (4)	2 (1.6)	0.268

Data are expressed as mean ± standard deviation, numbers (%) or median (interquartile range)

*T2DM* diabetes mellitus, *AMI* acute myocardial infarction, *BMI* body mass index, *Cr* creatinine, *TC* total cholesterol, *TG* triglyceride, *HDL-C* high-density lipoprotein cholesterol, *LDL-C* low-density lipoprotein cholesterol, *Hs-CRP* hypersensitive C-reactive protein, *HbA1c* hemoglobin A1c, *CK-MB* creatine kinase isoenzyme, *cTNI* cardiac troponin I, *ACEI/ARB* angiotensin-converting enzyme inhibitors/angiotensin receptor blocker, *DPP-4* dipeptidyl peptidase-IV

**Fig. 6:**
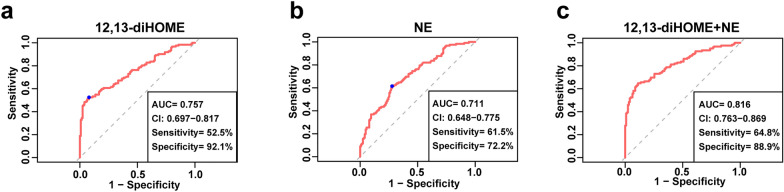
ROC curve analysis of the biomarker-based diagnostic model in distinguishing T2DM + AMI groups from T2DM patients. **a** 12,13-diHOME; **b** NE; **c** 12,13-diHOME + NE. N = 122 in T2DM + AMI; *NE* norepinephrine, *AUC* area under curve, *CI* confidence interval

Based on the ELISA results of the validation groups, which showed similar trends to the metabolomics results, we selected 12–13-diHOME and NE as biomarkers, considering that the levels of 12,13-diHOME and NE might play an essential role in the development of AMI in the T2DM population. In the T2DM + AMI group, 12,13-diHOME and NE showed a weak positive correlation with TNT at admission and no significant correlation with other clinical indicators (Additional file 5). In both T2DM group and T2DM + AMI group, 12,13-diHOME was not significantly correlated with NE (Additional file 6). We then analyzed 12,13-diHOME and NE levels using a multivariable logistic regression model along with clinically relevant baseline covariates (sex, history of smoking and hypertension, creatinine, TC, HDL-C, LDL-C, Hs-CRP, HbA1c, fasting blood glucose, prior treatment with statins, ACEI/ARB, and metformin), which were significantly different between the T2DM group and T2DM + AMI group. At the same time, we also corrected the age variable in the multivariable logistic regression model. The results showed that 12,13-diHOME (OR, 1.456; 95% CI 1.148–1.791) and NE (OR, 9.787; 95% CI 2.412–39.723) were independent risk factors for the development of AMI in patients with diabetes (Table 3). ROC curve analyses were used to further investigate the clinical diagnostic capability of the candidate metabolites. The AUC of 12,13-diHOME and NE were 0.757 and 0.711. The sensitivity was 52.5% and 61.5%, and the specificities were 92.1% and 72.2%, respectively (Fig. 6a and b). In addition, the combination of 12,13-diHOME and NE significantly improved the AUC to 0.816 (95% CI0.763–0.869; *P* < 0.001) (Fig. 6c). These results demonstrated that the combined model was reliable and could be applied to differentiate the occurrence of AMI in patients with T2DM.

**Table 3:** Univariate and multivariate logistic regression analyses of independent risk factors for development AMI in diabetic patients

	Univariate		Multivariate	
	OR (95% CI)	*P* value	Adjusted OR (95% CI)	*P* value
12–13-diHOME	1.414 (1.260–1.586)	< 0.001	1.456 (1.184–1.791)	< 0.001
Norepinephrine	5.427 (2.773–10.622)	< 0.001	9.787 (2.412–39.723)	0.001
Age	1.006 (0.981–1.032)	0.646		
Male gender	3.596 (2.110–6.127)	< 0.001		
Smoking	0.147 (0.083–0.262)	< 0.001	0.110 (0.021–0.583)	0.009
Hypertension	0.282 (0.167–0.476)	0.001	0.065 (0.016–0.265)	< 0.001
Cr, umol/L	1.020 (1.007–1.032)	0.002		
TC, mmol/L	1.588 (1.221–2.065)	0.001		
HDL-C, mmol/L	0.250 (0.081–0.770)	0.016		
LDL-C, mmol/L	2.182 (1.497–3.181)	< 0.001		
Hs-CRP, mg/L	1.101 (1.065–1.138)	< 0.001	1.106 (1.046–1.169)	< 0.001
HbA1c	1.720 (1.394–2.121)	< 0.001		
Fasting glucose, mmol/L	1.520 (1.332–1.734)	< 0.001	1.388 (1.053–1.829)	0.02
Statin	5.093 (2.533–10.239)	< 0.001		
ACEI/ARB	2.408 (1.424–4.072)	0.001	10.758 (2.454–47.171)	0.002
Diuretics	2.695 (1.612–4.504)	< 0.001	8.248 (2.362–28.799)	0.001

*OR* odds ratio

## Discussion

To the best of our knowledge, this is the first study to elucidate serum-specific metabolites associated with the occurrence of AMI in T2DM patients through an untargeted metabolomics approach. We identified 146 differentially-expressed serum metabolites among healthy controls and T2DM patients with and without an initial AMI. There were significant differences in 16 specific metabolites between T2DM patients with and without AMI. Among them, 12,13-diHOME and NE were markedly and independently associated with AMI onset. These findings provide new insights into the potential mechanisms by which 12,13-diHOME and NE could contribute to the development of AMI in the T2DM population.

Metabolites are particularly attractive as biomarkers in metabolic diseases because they frequently participate in disease pathways; their accumulation or deficiency can signal the presence of a disease. In this study, amino acids and lipids were the primary serum-specific differential metabolites between T2DM patients with and without AMI. This is consistent with the results of a previous study [9]. These are intermediate metabolites of carbohydrate, lipid, and amino acid-altered metabolism; they influence gluconeogenesis, glycolysis, lipolysis, the tricarboxylic acid cycle, and proteolytic pathways [9]. All of these processes are altered in the pathogenesis of T2DM, making amino acids and lipids promising candidate biomarkers for T2DM. In this study, we found that 12,13-diHOME and NE belong to lipid and amino acid classes, respectively. In addition, they were strongly and significantly associated with the onset of AMI in T2DM and showed great potential as specific serum biomarkers.

12,13-diHOME is a product of linoleic fatty acid through a reaction catalyzed by cyp450 epoxygenase and epoxide hydrolase [10]; it is a cold-induced oxylipin in mouse and human circulation. It is involved in thermogenesis and lipolysis. It partially improves glucose tolerance in the body, and is primarily produced by brown adipose tissue (BAT) under exposure to cold or exercise. 12,13-diHOME improves fat metabolism by inducing the transport and oligomerization of fatty acid transporter protein 1 (FATP1) and cluster of differentiation 36 (CD36) to increase free fatty acid uptake into BAT or skeletal muscle [11, 12]. Therefore, 12,13-diHOME or a functional analog could offer potential therapeutic strategies for metabolic disorders. However, 12,13-diHOME does not affect glucose uptake in some cells [12]. A Mendelian randomization analyses in a cohort of 2248 healthy participants indicated that genetically determined higher BMI, fasting hyperinsulinemia, and elevated lipid levels were not associated with changes in plasma 12,13-diHOME concentrations. In addition, there were no significant associations between 12,13-diHOME and HDL cholesterol and/or fasting glucose levels [13]. Similar to that in earlier reports, circulating 12,13-diHOME levels were not correlated with BMI, lipid levels, fasting glucose, or HbA1c in T2DM patients with AMI in this study. Although previous studies have shown that increased BAT activity is associated with lower blood glucose levels in humans, acute 12,13-diHOME treatment of mice in vivo increased skeletal muscle fatty acid up-take, but not glucose uptake [12, 14]. Consistent with our results, in this study about exercise-induced 12,13-diHOME, there was no correlation between 12,13-diHOME and fasting glucose concentrations [12]. Therefore, whether 12,13-diHOME can improve glucose metabolism in human beings requires more subsequent studies. Studies on 12,13-diHOME in the diabetic population are limited. In T2DM, serum 12,13-diHOME was positively correlated with C-peptide, fasting insulin, and evaluation of the homeostasis model of insulin resistance (HOMA-IR) and was negatively correlated with HbA1c [15]. These data could not determine the relationship between plasma levels of 12,13-diHOME and glucose metabolism, as this oxylipin is expected to be negatively correlated with serum insulin. This is the first study to show that the 12,13-diHOME level was similar between healthy controls and T2DM patients without CVD, but was significantly increased in T2DM patients with AMI, based on the untargeted metabolomics and ELISA results.

12,13-diHOME exhibits both beneficial and detrimental effects on the cardiovascular system. In a cohort of patients with heart disease, decreased levels of 12, 13-diHOME were correlated with lower ejection fraction. In addition, 12, 13-diHOME improved the in vivo cardiac hemodynamics by increasing cardiomyocyte contraction, relaxation, and mitochondrial respiration through activation of NOS1 [16]. However, this cohort has several limitations: a very small sample including only nine patients, predominantly male. In addition, the effect of 12,13-diHOME on cardiac function is contradictory. In murine hearts exposed to 12,13-diHOME after 20 min of ischemia, there was a decrease in post-ischemic functional recovery and inhibition of soluble epoxide hydrolase (sEH), an enzyme that produces 12,13-diHOME, preventing the harmful effects of acute cardiac ischemia [17]. In our validation phase study, 12,13-diHOME, an independent risk factor for the onset of AMI in patients with diabetes, showed a solid clinical identification capability. Therefore,12,13-diHOME could play a critical role in the development of AMI in patients with diabetes. To date, the underlying mechanisms remain unclear. Intra-abdominal treatment of mice with 12,13-diHOME increases pulmonary inflammation and decreases the number of regulatory T (Treg) cells in the lungs. Treatment of human dendritic cells with 12,13-diHOME alters the expression of PPARγ-regulated genes and reduces the secretion of anti-inflammatory cytokines and the number of Treg cells in vitro [18]. Tregs are a subset of T-cells with an immunomodulatory function; they can stabilize atherosclerotic plaques and prevent the development of AMI by reducing the production of inflammatory cytokines, inhibiting the expression of matrix metalloproteinase (MMP)-2 and MMP-9, increasing the expression of prolyl-4-hydroxylase α1, and suppressing the migration and adhesion of mature DCs [19]. Therefore, 12,13-diHOME could contribute to the progression of atherosclerosis and AMI in T2DM by decreasing Treg levels and inhibiting the secretion of anti-inflammatory cytokine. This study provides novel insights and confirms the above hypothesis through elucidating the significantly elevated levels of 12,13-diHOME in T2DM patients with AMI.

In addition to elevated 12,13-diHOME levels, NE levels were significantly higher in T2DM patients with AMI compared to that in those without AMI. NE is the primary neurotransmitter released from the postganglionic sympathetic neurons in peripheral tissues. It is both a neurotransmitter and a hormone, and is chemically a phenol. NE increases heart rate, cardiac contractility, vascular tone, and renin-angiotensin system activity in the periphery by activating adrenergic receptors [20]. The secretion of norepinephrine increases hepatic gluconeogenesis, inhibiting glucose entering muscle and adipose tissue cells and raising blood glucose. Overexpression of the primary mediator of the inhibitory effects of norepinephrine can cause a progressive loss of β-cell function leading to T2DM [21]. These studies suggested that the high blood glucose is one of the physiopathologic mechanisms for NE to result in myocardial infarction. Pharmacological inhibition of the increased central NE outflow has a positive influence on body weight and glucose and lipid metabolism [22], suggesting that inhibiting NE release or production is promising for alleviating the development of T2DM. NE is involved in the development of cardiovascular diseases. Chronic exposure to psychosocial stress adversely affects cardiovascular health. The locus coeruleus (LC)-NE system contributes to stress-induced cardiovascular disease [23]. A small increase in concentration leads to myocardial damage through direct catecholamine toxicity, epicardial and microvascular coronary vasoconstriction and/or spasm, and improved cardiac workload [24]. NE can exacerbate the inflammatory response and post-infarction ventricular remodeling [25].

Similar to 12,13-diHOME, NE was explicitly upregulated in T2DM patients with AMI. NE is associated with the onset of AMI through the activation of adrenergic signaling in heart tissues. NE treatment of mice for 30 min, which mimics sympathetic activation, induces 12,13-diHOME production. 12,13-diHOME could be a potential product of sympathetic activation [11]. However, a positive correlation between 12,13-diHOME and NE was not observed in T2DM patients with AMI, which could be attributed to the multiple sources of 12,13-diHOME. In the validation cohort, these two metabolites were positively correlated with TNT levels during admission, suggesting that 12,13-diHOME and NE could play a role in the development of AMI in T2DM patients. The relationships between 12,13-diHOME, NE, and other cardiac enzymes would require a larger validation population.

This study has certain limitations. First, it used a single cohort with a relatively small sample size for the untargeted metabolomics analysis; therefore, the results and conclusions drawn for these metabolites cannot be generalized to other populations and should be interpreted with caution. A wider multicenter replication is required to verify the findings of this study. Second, 12,13-diHOME and NE levels may be influenced by physical exercise. All blood samples in this study were collected at admission; the level of physical activity a few hours before blood collection should have been recorded and analyzed, as an acute exercise spurt increases serum 12,13-diHOME and NE levels in humans. Third, given this study's cross-sectional design, combining our present study's results with the previous published research does not provide a causal relationship between metabolites and the occurrence of AMI in the diabetic population. Therefore, a prospective study is needed to further validate the role of 12,13-diHOME and NE in T2DM patients with AMI. Finally, gene pleiotropy could not be avoided. Functional genomic studies for these two metabolites, with larger sample sizes, are required. Their exact roles in the molecular etiology and physiology should be elucidated through functional studies.

## Conclusions

16 specific serum metabolites were differentially expressed between T2DM patients without CVD and those with initial AMI through untargeted metabolomics. Moreover, amino acid and lipid pathways were identified to be the mainly involved pathways. Notably, serum 12,13-diHOME and NE metabolites were associated with the occurrence of AMI in patients with T2DM, indicating that these circulating metabolites might be used for identifying the population at a higher risk of AMI. These findings are important to expand the mechanism of development AMI in the diabetic population and to provide new therapeutic targets to reduce the incidence of AMI in the future.

## Supplementary Information

**Additional file 1. **The detailed description of LC–MS methodology and quality control results in this untargeted metabonomic.

**Additional file 2. **The detailed information of all the identified serum metabolite among T2DM patients with or without AMI detected by untargeted metabonomic. N = 222. D, Type II diabetes. A, acute myocardial infarction. C, healthy control. Screening methods: In OPLS-DA model, Variable important in projection (VIP) > 1, *P* < 0.05. T2DM, Type II diabetes; AMI, Acute myocardial infarction.

**Additional file 3. **The detailed information of 146’s serum differential metabolites among T2DM patients with or without AMI detected by untargeted metabonomic. Screening methods: In OPLS-DA model, Variable important in projection (VIP) > 1, *P* < 0.05. T2DM, Type II diabetes; AMI, Acute myocardial infarction.

**Additional file 4. **The detailed information of 16’s serum specific differential metabolites between T2DM group and T2DM + AMI group detected by untargeted metabonomic.

**Additional file 5. **The correlation analysis of clinical indicators with 12,13-diHOME and NE in T2DM + AMI patients.

**Additional file 6. **The correlation analysis of serum levels of 12,13-diHOME and NE in T2DM and T2DM + AMI patients.

## Data Availability

The data analyzed in this study can be obtained from the corresponding author with a reasonable request.

## Abbreviations

AMI: Acute myocardial infarction
T2DM: Type 2 diabetes mellitus
IDF: International Diabetes Federation
CVD: Cardiovascular disease
12,13-diHOME: 12,13-Dihydroxy-9Z-octadecenoic acid
ES: Estrone sulfate
NE: Noradrenaline
STEMI: ST-elevation myocardial infarction
RBG: Random blood glucose
OGTT: Oral glucose tolerance test
LC–MS: Liquid chromatography-mass spectrometry
KEGG: Kyoto encyclopedia of genes and genomes
ELISA: Enzyme linked immunosorbent assay
SD: Standard deviation
LSD: Least significant difference
ROC: Receiver operating characteristic
AUC: Area under the curve
OPLS-DA: Orthogonal projection to latent structure-discriminant analysis
PCA: Principal component analysis
VIP: Variable importance in the point
OR: Odds ratios
CI: Confidence intervals
QC: Quality control
BMI: Body mass index
Cr: Creatinine
TC: Total cholesterol
TG: Triglyceride
LDL-C: Low-density lipoprotein cholesterol
HDL-C: High-density lipoprotein cholesterol
Hs-CRP: Hypersensitive C-reactive protein
HbA1c: Hemoglobin A1c
CK-MB: Creatine kinase isoenzyme
cTNI: Cardiac troponin I
cTnT: Cardiac troponin T
ACEI/ARB: Angiotensin-converting enzyme inhibitors/angiotensin receptor blocker
DPP-4: Dipeptidyl peptidase-IV
BAT: Brown adipose tissue
FATP1: Fatty acid transporter protein 1
CD36: Cluster of differentiation 36
sEH: Soluble epoxide hydrolase
MMP: Matrix metalloproteinase
