# Supplementary material for: 12,13-diHOME and noradrenaline are associated with the occurrence of acute myocardial infarction in patients with type 2 diabetes mellitus
Source: Diabetol Metab Syndr. 2023 Jun 29;15:142. doi: 10.1186/s13098-023-01115-9 (PMC10308632; doi:10.1186/s13098-023-01115-9)
Supplement: Supplementary file 1 — Additional file 1. The detailed description of LC–MS methodology and quality control results in this untargeted metabonomic. [file 13098_2023_1115_MOESM1_ESM.docx]

Additional file 1. The detailed description of LC-MS methodology and quality control results in this untargeted metabonomic.

The LC-MS sample preparation process:

Before metabolomics analysis, serum samples were thawed at 4℃. Then we add 100 µL of mixed internal standard solution and 400 µL of methanol (-20℃). After vortex and centrifugation, the supernatant was concentrated and dried under vacuum, and then dissolved with methanol. The re-obtained supernatant was used for LC/MS analysis. In addition, 20 ul of each sample to be tested was mixed into quality control (QC) samples.

Additional description of the LC-MS method:

The resolution volume, isotope internal standard identification and acquisition of standard curve solution have been mentioned in the original manuscript. The mobile phase was composed of 0.1% formic acid in water (A2)and 0.1% formic acid in acetonitrile (B2) or 5 mM ammonium formate in water (A3) and acetonitrile (B3) at a flow rate of 0.25 mL/min. Injection of 2-μL of each sample was done after equilibration. An increasing linear gradient of solvent B2/B3 (v/v) was used as follows: 0-1 min, 2% B2/B3; 1-9 min, 2%-50% B2/B3; 9-12 min, 50%-98% B2/B3; 12-13.5 min, 98% B2/B3; 13.5-14 min, 98%-2% B2/B3 and 14-20 min, 2% B2-positive model (14-17 min, 2% B3-negative model).


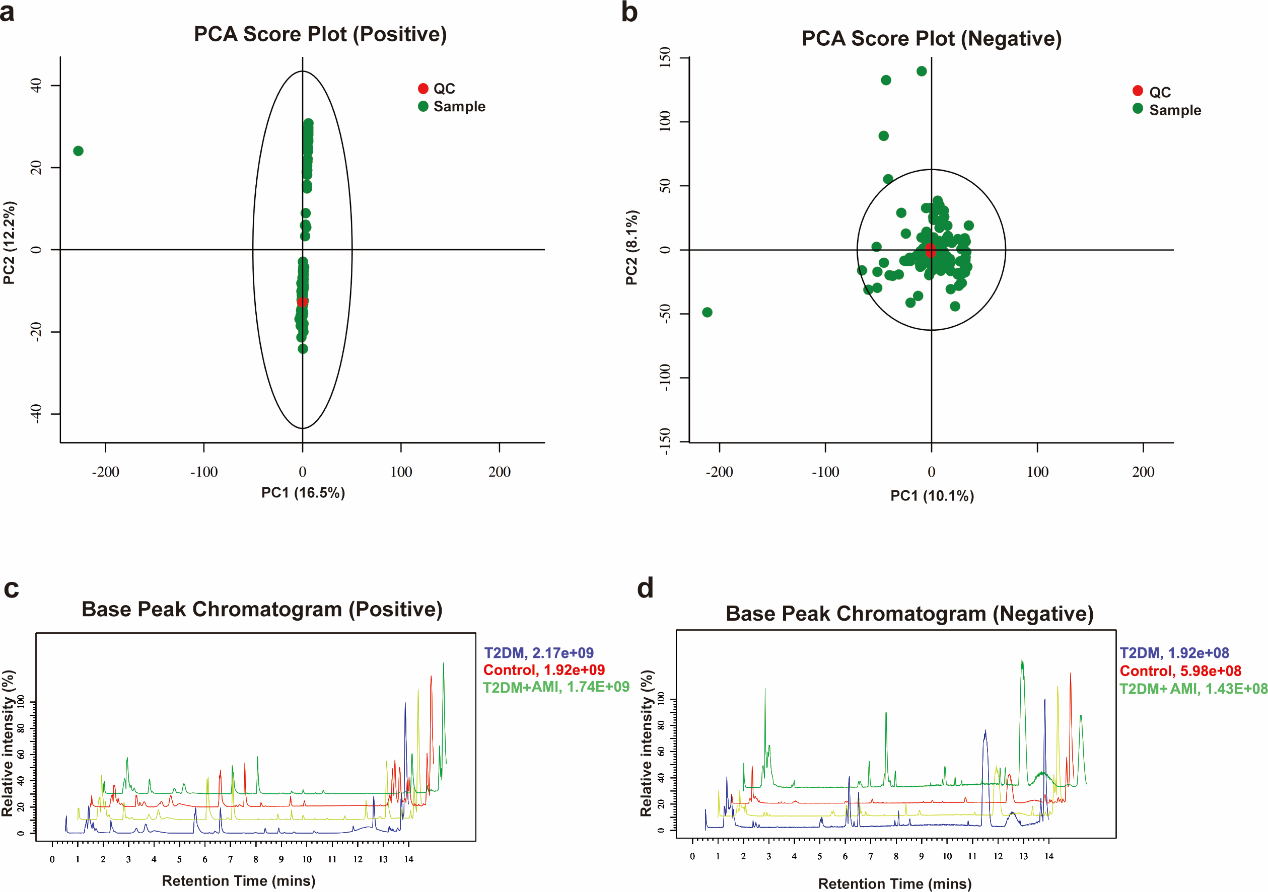


Figure. The quality control result in this untargeted metabonomic. a and b, The PCA score plots of serum metabolites and quality control either in positive or negative model in the untargeted metabonomic. QC, quality control, red circle. Samples, green circles. c and d, the base peak chromatogram of samples in different groups either in positive or negative model in the untargeted metabonomic. T2DM, Type II diabetes; AMI, Acute myocardial infarction.
