# Supplementary material for: 12,13-diHOME and noradrenaline are associated with the occurrence of acute myocardial infarction in patients with type 2 diabetes mellitus
Source: Diabetol Metab Syndr. 2023 Jun 29;15:142. doi: 10.1186/s13098-023-01115-9 (PMC10308632; doi:10.1186/s13098-023-01115-9)
Supplement: Supplementary file 5 — Additional file 5. The correlation analysis of clinical indicators with 12,13-diHOME and NE in T2DM + AMI patients. [file 13098_2023_1115_MOESM5_ESM.docx]

Additional file 5. The correlation analysis of clinical indicators with 12,13-diHOME and NE in T2DM+AMI patients

|  | 12,13-diHOME | NE |
| --- | --- | --- |
| Age,year | 0.168 | 0.072 |
| BMI,Kg/m^2^ | -0.128 | -0.027 |
| Duration of diabetes,years | 0.146 | 0.090 |
| Cr,mmol/L | 0.043 | 0.034 |
| TC,mmol/L | -0.126 | -0.127 |
| TG,mmol/L | -0.105 | -0.104 |
| HDL-C,mmol/L | -0.062 | 0.052 |
| LDL-C,mmol/L | -0.102 | -0.117 |
| Apo-A1,mmol/L | -0.102 | -0.093 |
| Apo-B,mmol/L | -0.134 | -0.144 |
| Hs-CRP,mg/L | 0.034 | 0.113 |
| HbA1c,% | -0.037 | 0.026 |
| CK-MB,ng/ml | -0.121 | 0.027 |
| cTNI,ng/ml | -0.083 | 0.043 |
| cTNT,ng/ml | **0.207^a^** | 0.181^a^ |

Pearson correlation analysis was used between the two groups of variables, a) p<0.05; T2DM, Type II diabetes mellitus; AMI, acute myocardial infarction; NE, Norepinephrine; BMI, body mass index; Cr, creatinine; TC, total cholesterol; TG, triglyceride; HDL-C, high-density lipoprotein cholesterol; LDL-C, low-density lipoprotein cholesterol; Hs-CRP, hypersensitive C-reactive protein; HbA1c, hemoglobin A1c; CK-MB, creatine kinase isoenzyme; cTNI, cardiac troponin I; TNT, cardiac troponin T.
