# Supplementary material for: 12,13-diHOME and noradrenaline are associated with the occurrence of acute myocardial infarction in patients with type 2 diabetes mellitus
Source: Diabetol Metab Syndr. 2023 Jun 29;15:142. doi: 10.1186/s13098-023-01115-9 (PMC10308632; doi:10.1186/s13098-023-01115-9)
Supplement: Supplementary file 6 — Additional file 6. The correlation analysis of serum levels of 12,13-diHOME and NE in T2DM and T2DM + AMI patients. [file 13098_2023_1115_MOESM6_ESM.docx]

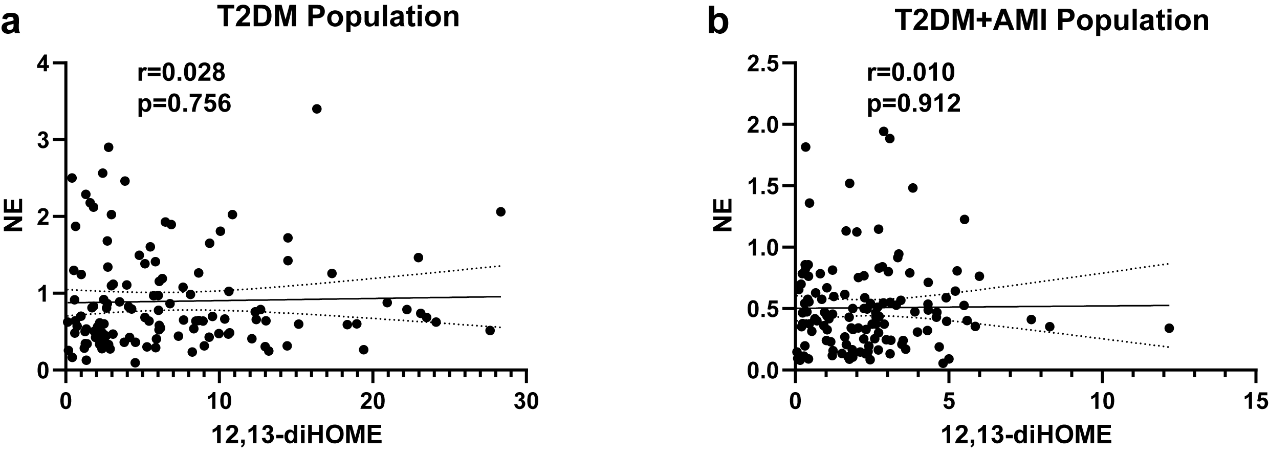


**Additional file 6. The correlation analysis of serum levels of 12,13-diHOME and NE in DM and T2DM+AMI patients.** The serum concentrations of 12,13-diHOME and NE were measured by ELISA in T2DM patients with or without AMI. N=122 in T2DM+AMI, N=126 in T2DM, 12,13-diHOME, 12,13-dihydroxy-9Z-octadecenoic acid. NE, Norepinephrine. T2DM, Type II diabetes; AMI, [Acute myocardial infarction](javascript:;). *Pearson* correlation analysis.
